# Towards a more holistic research approach to plant conservation: the case of rare plants on oceanic islands

**DOI:** 10.1093/aobpla/plv066

**Published:** 2015-06-11

**Authors:** Luís Silva, Elisabete Furtado Dias, Julie Sardos, Eduardo Brito Azevedo, Hanno Schaefer, Mónica Moura

**Affiliations:** 1 InBIO, Rede de Investigação em Biodiversidade, Laboratório Associado, CIBO, Centro de Investigação em Biodiversidade e Recursos Genéticos, Polo-Açores, Departamento de Biologia, Universidade dos Açores, 9501-801 Ponta Delgada, Açores, Portugal; 2 Bioversity-France, Parc Scientifique Agropolis II, 34397 Montpellier Cedex 5, France; 3 Research Center for Climate, Meteorology and Global Change (CMMG - CITA-A), Departamento de Ciências Agrárias, Universidade dos Açores, Angra do Heroísmo, Açores, Portugal; 4 Plant Biodiversity Research, Technische Universität München, D-85354 Freising, Germany

**Keywords:** Azores, conservation, germination, population genetics, species distribution models, threats, *Veronica dabneyi*

## Abstract

Research dedicated to rare endemic plants is usually focused on one given aspect. However, holistic studies, addressing several key issues, might be more useful, supporting management programmes while unravelling basic knowledge about ecological and population-level processes. A more comprehensive approach to research is proposed, encompassing: phylogenetics/systematics, pollination biology and seed dispersal, propagation, population genetics, species distribution models (SDMs), threats and monitoring. We present a holistic study dedicated to *Veronica dabneyi* Hochst. ex Seub., an endangered chamaephyte endemic to the Azores. *Veronica dabneyi* was mainly found associated with other endemic taxa; however, invasive plants were also present and together with introduced cattle, goats and rabbits are a major threat. Most populations grow at somewhat rocky and steep locations that appeared to work as refuges. Seed set in the wild was generally high and recruitment of young plants from seed seemed to be frequent. In the laboratory, it was possible to germinate and fully develop *V. dabneyi* seedlings, which were planted at their site of origin. No dormancy was detected and time for 50 % germination was affected by incubation temperature. Eight new microsatellite markers were applied to 72 individuals from 7 sites. A considerable degree of admixture was found between samples from the two islands Flores and Corvo, with 98 % of the genetic variability allocated within populations. Levels of heterozygosity were high and no evidence of inbreeding was found. Species distribution models based on climatic and topographic variables allowed the estimation of the potential distribution of *V. dabneyi* on Flores and Corvo using ecological niche factor analysis and Maxent. The inclusion of land-use variables only slightly increased the information explained by the models. Projection of the expected habitat in Faial largely coincided with the only historic record of *V. dabneyi* on that island. This research could be the basis for the design of a recovery plan, showing the pertinence of more holistic research approaches to plant conservation.

## Abstract (precis)

Research dedicated to rare endemic plants is usually focused on one given aspect. However, holistic studies, addressing several key issues, might be more useful, supporting management programs, while unravelling basic knowledge about ecological and population level processes. A more comprehensive approach to research is proposed, encompassing: phylogenetics/systematics, pollination biology and seed dispersal, propagation, population genetics, species distribution models, threats and monitoring. We applied this research approach to *Veronica dabneyi*, an endangered chamaephyte endemic to the Azores, showing how it could support more effective recovery plans.

## Introduction

Island endemic plants are among the most threatened group of organisms worldwide (Caujapé-Castells *et al*. 2010). Research dedicated to rare endemic plants is usually focused on one or few aspects, such as conservation genetics, propagation or distribution (e.g. Dubuis *et al*. 2013; Evans *et al*. 2014; Mir *et al*. 2014). Multidisciplinary studies, addressing several key issues, are much more useful but still the exception (e.g. Menges 1990; Halbur *et al*. 2014). They have the potential to provide science-based evidence to management or recovery programmes while at the same time unravelling basic knowledge about the population processes and the ecology of endangered species. We propose a general framework towards more holistic conservation research, particularly when devoted to rare plants on oceanic islands. We suggest that such approach should include the following critical areas: (i) phylogenetics/systematics (i.e. DNA sequences and morphology) to determine origin and close relatives as well as the existence of unaccounted taxa (Bateman *et al*. 2013); (ii) population genetics to estimate genetic structure and diversity (Dias *et al*. 2014), identify possible cases of inbreeding depression (Li *et al*. 2012) and ensure adequate provenance of propagation material (Silva *et al*. 2011; Hancock and Hughes 2014); (iii) germination biology and propagation methods to identify possible biological constraints (Beaune *et al*. 2013) and support the species recovery (Pence 2013); (iv) pollination biology and research on dispersal mechanisms to identify possible biological/ecological constraints (Ollerton *et al*. 2011; Rodríguez *et al*. 2015); (v) the identification of threats, including invasive plants (Foxcroft *et al*. 2013) and animals, particularly herbivores (Donlan *et al*. 2003; Garzón-Machado *et al*. 2010, Barrios-Garcia *et al*. 2014); (vi) species distribution models (SDMs) to determine environmental constraints and potentially favourable areas, possible impacts of climate change and of other anthropogenic alterations (Costa *et al.* 2012, 2013*a*; Marcer *et al*. 2013) and (vii) long-term monitoring (minimum 10 years, depending on the duration of the species’ generation time) to evaluate population fluctuations and the effect of management actions (Godefroid *et al*. 2011). Besides addressing those biological issues, conservation research should also integrate possible societal aspects (i.e. the different stakeholders that will have a direct or indirect role in the conservation process).

Here we present an example of a multidisciplinary study, using *Veronica dabneyi* Hochst. ex Seub. (Plantaginaceae Juss.), known as ‘veronica’ or ‘Azorean speedwell’, a rare chamaephyte (subshrub) endemic to the Azores islands.

The genus *Veronica* L. is the largest of Plantaginaceae with ∼450 species. It is distributed worldwide, with a large range of life forms from diverse habitats (Albach *et al*. 2005). Based on morphological affinities and preliminary molecular data, *V. dabneyi* is a close relative of *V. officinalis* L., belonging to the subgenus *Veronica* L., a clade also including *V. alpina* L. and *V. montana* L. (Albach and Meudt 2010).

*Veronica dabneyi* was first described by Karl C.F. Hochstetter in 1838, after a visit to Faial Island. Since the plant was cultivated as ornamental in the garden of the American consul, Charles William Dabney in Horta (Faial), Hochstetter attributed the specific name presently used. Seubert (1844) published the description with drawings in his *Flora Azorica*. Later, Watson (1870) reported *V. dabneyi* for São Miguel, Faial and Corvo Islands, without specifying the exact places where the species was found. Cunha and Sobrinho (1939) collected *V. dabneyi* in Faial, at the inner side of the Caldeira summit, the only record published during the 20th century (specimen in LISU, seen by HS). The species was later cited as extinct by Catarino *et al*. (2001). However, it turned out that the species was still extant in inaccessible parts of the western islands (Pereira *et al*. 2002). Those authors observed a number of mature plants on Flores and Corvo, but noticed that young plants were rare and seed production was low, probably due to predation by goats and rabbits, and to the occurrence of environmental disturbance (e.g. landslides, trampling by cattle). The species was found to be associated with a vegetation type described by Sjögren (1973) as *Festucetum jubatae*, dominated by *Festuca francoi* Fern.Prieto, C.Aguiar, E.Dias & M.I.Gut (Poaceae Barnhart). The conservation status was reevaluated according to the International Union for Conservation of Nature criteria and it was classified as extinct in Faial and as critically endangered in Flores and Corvo (Pereira *et al*. 2002), based on an area of occurrence of 63 km^2^, with 16 subpopulations in Flores, and one in Corvo. In general, the populations only included a few individuals, while no individuals were found in the wild at Faial. *Veronica dabneyi* was thus classified as extinct in Faial and as critically endangered in Flores and Corvo. In a global analysis of the conservation status of Azorean indigenous species, Silva *et al*. (2009) suggested possible natural threats (storms, strong wind, landslides), biological limitations (isolation of populations), as well as threats of human origin (expansion of invasive plants and introduced herbivores, changes in land use) as causes of species decline.

Two aspects that should be addressed as important contributions to the species long-term survival are estimates of genetic diversity and population genetic structure. These are crucial for any sort of recovery plan for endangered plants, particularly in islands, where genetic diversity has been often expected to be lower than in mainland populations (e.g. Caujapé-Castells *et al*. 2008; Silva *et al*. 2011; Martins *et al*. 2013; Moreira *et al*. 2013; Moura *et al*. 2013; Dias *et al*. 2014).

Propagation measures, particularly those allowing the maintenance of the genetic variability, such as seed germination, are also critical in the recovery of endangered plants (e.g. Moura and Silva 2010; Martins *et al*. 2012; Moreira *et al*. 2012).

Modelling is nowadays a common approach for predicting species distributions. This is based on statistically or theoretically derived response surfaces that link the known distribution of a species to the pertinent environmental descriptors, allowing to estimate its potential distribution, as well as to determine the environmental factors limiting its range (Guisan and Zimmermann 2000). Species distribution modelling has been used in a wide range of applications (Elith and Leathwick 2009), including the evaluation and management of endangered species (e.g. Engler *et al*. 2004; Marcer *et al*. 2013).

Integrating the above aspects in the design of a multidisciplinary research programme is an example of a more holistic approach to be applied in plant conservation, as a basis for recovery plans. Here, we reanalyse the available data for *V. dabneyi*, and provide new information on germination rate and population genetic structure and diversity. We also analyse species distribution in order to understand what ecological factors might be constraining it. Due to the small size of the populations, and to the degree of isolation and fragmentation, we expect to find reduced levels of seed set, comparably low levels of genetic diversity and some degree of differentiation between the studied populations. Because it is rare, we expect the species to have a relatively restricted ecological niche and high levels of ecological specialization.

## Methods

### Sampling

#### *Veronica dabneyi* populations

**Figure 1.**
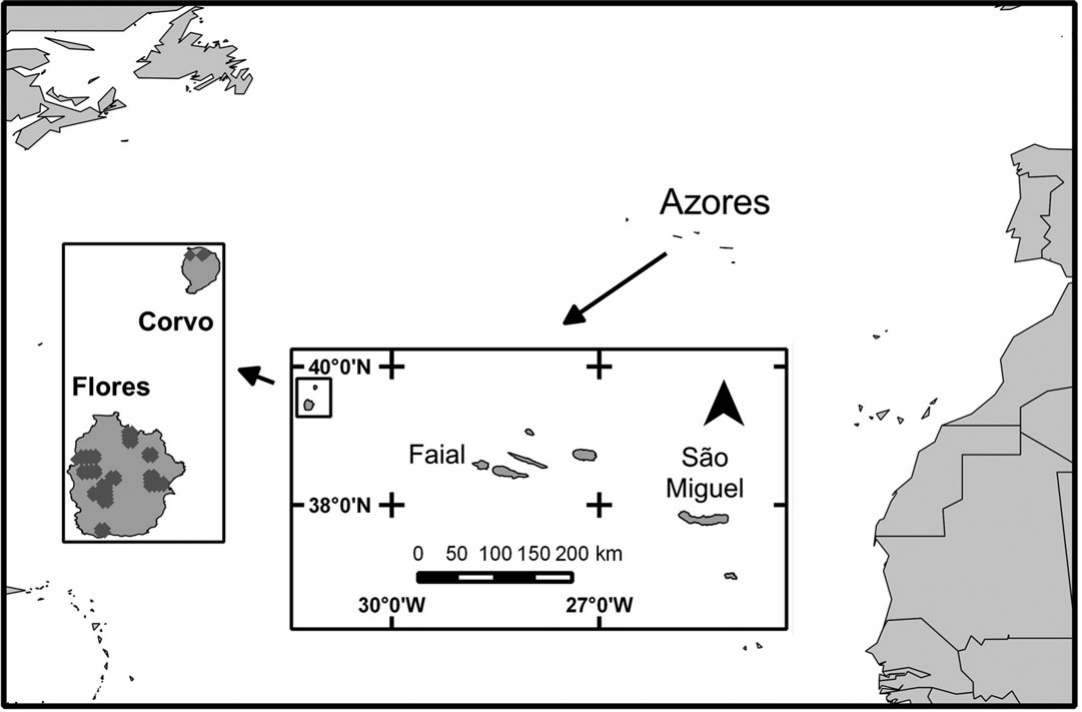
The location of the Azores Archipelago in the North Atlantic Ocean, and recorded presence of *Veronica dabneyi* in Flores and Corvo islands.

*Veronica dabneyi* was searched in areas with potentially suitable habitat on the islands of Corvo, Flores and Faial (Fig. 1), between the years 2000 and 2014. The search was not successful on Faial but on Flores and Corvo a total of seven (sub)populations were found. All cited locations here are recorded in the Atlantis database of the ‘Azorean Biodiversity Portal’ (Borges *et al*. 2010), including data from Pereira *et al*. (2002) and Schaefer (2003).

#### Demographic data

Three populations were studied in detail: Miradouro Craveiro Lopes and Tapada da Forcada (Flores) and Madeira Seca (Corvo). Variables measured included: individual number and size, stem length, number of inflorescences and inflorescence height (measured in loco), number of fruits per inflorescence, number of seeds per fruit, seed diameter and weight, using a digital calliper and an electronic scale (measured/counted at the laboratory). Fruits were sampled in July 2008 from Miradouro Craveiro Lopes, and in June 2010 form Madeira Seca and Tapada da Forcada.

#### Associated flora and invasive species

Characterization of the associated flora was based on the work by Pereira *et al*. (2002) and on data collected by the authors in 2010, using a 1 m^2^ plot centred on *V. dabneyi* individuals or groups of individuals. The presence of plant invaders (see Silva *et al*. 2008) was recorded from the immediate surroundings of *V. dabneyi* populations. The presence of cattle, goats and rabbits was confirmed through direct visual observation of the animals or by the presence of characteristic faeces.

#### Plant material for genetic analysis

In 2008 and 2010, a collection of leaf material was carried out to complement the samples already available at the DNA bank collection of the AZB herbarium (Biology Department, Azores University). Depending on leaf size, one or two leaves per individual were collected and immediately stored in a plastic bag with silica gel. After drying, the leaves were vacuum sealed and stored in folders. The number of individuals sampled per site varied from 10 to 30 individuals depending on the population size. In total, 72 individuals from 7 different sites were sampled (Fig. 1, Table 1). The plant material obtained from the F1 of Miradouro Craveiro Lopes (i.e. obtained from the germinated seedlings) was also used for comparison with the mother population.

**Table 1.** Location and number of samples of *V. dabneyi* for population genetic analysis in the Islands of Flores and Corvo (Azores). Voucher code for the material deposited at AZB (Herbarium Ruy Telles Palhinha), population code used in the study, local designation for each site, elevation (m above sea level), universal transverse mercator coordinates (*X*, *Y*; WGS84 25S) and number of samples per site (*N*). Three individuals resulting from seed germination were also included (i.e. F1 Miradouro Craveiro Lopes).

Island	Voucher	Code	Site	Elevation	*X*	*Y*	*N*
Flores	VD-FLML-001	VDFLML	Miradouro Craveiro Lopes	482	651 717	4 365 683	26
Flores	VD-FLTP-001	VDFLTP	Tapada da Forcada	490	654 964	4 373 203	12
Corvo	VD-COMS-005	VDCOMS	Madeira Seca	442	662 989	4 398 153	18
Corvo	VD-COMC-002	VDCOMC	Caldeirão I	600	662 653	4 397 918	4
Corvo	VDCO1A	VDCO1	Caldeirão II	569	662 640	4 397 900	3
Corvo	VDCO3A	VDCO3	Arribas do Caldeirão	255	661 310	4 397 900	3
Corvo	VDCO2A	VDCO2	Arribas	255	661 330	4 397 900	3

### Propagation

#### Seed germination

Fresh seeds collected in 2008 and 2010, as well as 2-year-old seeds stored at room temperature, were used for germination tests. Germination tests were done in Petri dishes using growth chambers with automatic temperature control (error margin of ∼1 °C) and a light period of 12 h per day, provided by six fluorescent lamps with a photosynthetic photon flux density (PPFD) of 19–22 μmol m^−2^ s^−1^. The chambers were set to the incubation temperatures of 25/20, 20/15, 15/10 and 10/5 °C, and the highest temperature coincided with the 12 h of the photophase. Germination proceeded in light or in darkness (Petri dish covered with aluminium foil) with three replicates per treatment and 17, 18 or 56 seeds per replicate, depending on the total number of seeds sampled at each site. Seeds were monitored daily and considered to be germinated when the radicle extruded. Seeds from the dark treatments were observed under a green light. Accumulated germination curves were adjusted to a Gompertz model to allow the calculation of the time, in days, necessary for 50 % seed germination (T50). The latter model has been successfully used to describe accumulated germination while allowing a biological interpretation (see Latera and Bazzalo 1999; Moura and Silva 2010).

#### Seedling growth and establishment

Germinated seedlings were planted in Jiffy^®^ peat pellets in a growth chamber with regulated temperature (20 °C) and photoperiod (12 h of photophase) for 2 months, and regularly watered to avoid substrate desiccation. The seedlings were transported to Flores Island and planted near the mother plants at Miradouro Craveiro Lopes, and were followed up every 6 months during 2 years.

### Genetic analysis

#### General DNA extraction

Deoxyribonucleic acid was extracted from dry leaves using a modified hexadecyl-trimethyl-ammonium bromide extraction method (Doyle and Dickson 1987) without the final ethanol wash. Deoxyribonucleic acid was then precipitated by adding 450 μL of isopropanol and re-suspended in 50 μL of pure water. The DNA quality and quantity were measured using a Nanodrop 2000 (Thermo Fisher Scientific) spectrophotometer. Samples were conserved at −20 °C until use.

#### Microsatellite development

Total DNA from fresh leaves of one individual of *V. dabneyi* was sent to the Savannah River Ecology Laboratory (University of Georgia, USA), where the enrichment procedure described in Glenn and Schable (2005), with the exceptions described in Lance *et al*. (2010), was followed for microsatellite isolation. CAP3 (Huang and Madan 1999) was used to assemble sequences at 98 % sequence identity using a minimal overlap of 75 bp. Search for microsatellite DNA loci was conducted using the programme MSATCOMMANDER version 0.8.1 (Faircloth 2008) and primers designed with Primer3 (Rozen and Skaletsky 2000). One primer from each pair was extended on the 5′-end with an engineered sequence (M13R tag 5′-GGAAACAGCTATGACCAT-3′) to enable the use of a third primer identical to the M13R (Schuelke 2000), and a GTTT ‘pigtail’ was added to the 5′-end of the untagged primer to facilitate accurate genotyping (Brownstein *et al*. 1996). Out of the 202 sequences of primer pairs provided by the Savannah River Ecology Lab, we selected 24 primer pairs, 12 with expected polymerase chain reaction (PCR) products ranging between 100 and 200 bp (A series) and 12 exhibiting expected PCR products ranging between 200 and 300 bp (B series) to allow later the multiloading of PCR products. All the primer pairs (with the tag sequence included) were selected on criteria of non-complementarities within and between primers, low secondary structures and 3′-end instability (Rychlik 1995).

#### Microsatellite selection and full-scale genotyping

All 24 primer pairs were tested on eight samples of *V. dabneyi* using a unlabelled tag primer (M13R) in a final volume of 25 μL consisting of 25 ng of DNA, 75 μg mL^−1^ BSA, 1× NH_4_ buffer, 2 mM MgCl_2_, 0.4 μM of untagged primer, 0.08 μM of tagged primer, 0.36 μM of Universal dyed M13R, 200 μM of dNTPs, 1 U of Immolase (Bioline) and using a Biometra TGradient thermocycler. Touchdown thermal cycling programmes (Don *et al*. 1991) encompassing a 10 °C span of annealing temperatures ranging between 63 and 53 °C were used for all loci. The PCR programme included the following steps: 95 °C for 7 min (hot start); 96 °C for 3 min; 20 cycles of 95 °C for 30 s, the highest annealing temperature of 63 °C (decreased by 0.5 °C per cycle) for 30 s, and 72 °C for 30 s; 20 cycles of 95 °C for 30 s, 53 °C for 30 s, and 72 °C for 30 s and finally 72 °C for 10 min for the final extension of the PCR products. Five microlitres of PCR products were then run on a 3.5 % agarose gel, stained with SafeView™ Classic Nucleic Acid Stain (ABM, Inc.) and visualized under UV to check for amplification, polymorphism and scorability of the bands. Ten primer pairs exhibited scorable amplified products of the expected length range and with at least two alleles.

After analysis of the quality of the PCR products obtained with the universal primer M13R, 10 primers with acceptable to high scorability were selected to run the complete study (Table 2). After optimization, the amplifications for the whole sample were performed using the protocol indicated above with the alterations presented in Table 3, and the M13R labelled either with PET, FAM, NED or VIC. Amplification products were diluted, multiloaded, run on an ABI-3130xl Genetic Analyzer and sized with LIZ500 size standard. The genotypes obtained were scored using the software GeneMarker V.1.97 Demo version (Softgenetics).

**Table 2.** Set of simple sequence repeats primers that showed scorable and polymorphic products for the analysis of *Veronica dabneyi* population genetic structure in the Azores.

Primer	Motif	Forward	Reverse
Vd2B04	AC(9)	GTTTAGTGACGAGGACATTGATTG	GGAAACAGCTATGACCATCCTTCTAACATCGCAAACTG
Vd2B09	AC(10)	GTTTGCACACTGAAGGGTATCAAC	GGAAACAGCTATGACCATAAATCGGTGAATGTTTGATC
Vd3A01	AAG(9)	GTTTGTGTTCAGCTTGGAATTGAG	GGAAACAGCTATGACCATCTCTTCGACCAAATTCTTG
Vd3A03	ATC(18)	GGAAACAGCTATGACCATAAGTTCTTGCTCTGCTTGTC	GTTTCTTGTAGCCCAGATTGAAAC
Vd3A05	ATC(14)	GGAAACAGCTATGACCATCTAAACTCCCTTTCACTGG	GTTTGCGTCGAAGTACAAGAACAG
Vd3B07	AAG(15)	GTTTAGCTCGGAAACTTTGTAATG	GGAAACAGCTATGACCATGCAATAAAGTGATTAAGTGG
Vd4A01	AATG(6)	GTTTCCCACTATCCAACCATAATC	GGAAACAGCTATGACCATAACTCAGCTCAGCGTGAC
Vd4B01	AAAC(11)	GGAAACAGCTATGACCATAACCACATCACTCCAAACAG	GTTTGACTGGGCTAGAGTTGTC
Vd4B04	ACAT(20)	GTTTAATCCATTGTGTGCAGTCTC	GGAAACAGCTATGACCATCACCTCCCACACTTAATC
Vd4A03	AAAC(6)	GGAAACAGCTATGACCATGCTTTAATTTGTGCGTATC	GTTTCCTATCCCTTAACCTTTCTTC

**Table 3.** Optimization conditions used in the full-scale genotyping of *Veronica dabneyi*.

Primer	Optimization
Vd2B04	0.36 µM VIC, 0.5 U immolase
Vd2B09	0.36 µM NED, 0.5 U immolase
Vd3A01	0.36 µM FAM, 0.5 U immolase
Vd3A03	0.04 µM tagged primer, 0.36 µM FAM, 0.5 U immolase
Vd3A05	0.04 µM tagged primer, 0.36 µM VIC, 0.5 U immolase
Vd3B07	0.04 µM tagged primer, 0.36 µM NED, 0.5 U immolase, 50 ng DNA
Vd4A01	0.04 µM tagged primer, 0.36 µM NED, 0.5 U immolase
Vd4B01	0.36 µM VIC, 0.5 U immolase
Vd4B04	0.2 µM tagged primer, 0.2 µM FAM, 0.75 U immolase, 50 ng DNA
Vd4A03	0.36 µM PET, 0.5 U Immolase

#### Analysis of genetic data

Population structure was analysed with GenAlEx 6.5 (Peakall and Smouse 2012), to obtain mean values per population of the total number of alleles, the number of alleles with a minimum allele frequency of 5 %, the number of effective alleles, the Shannon's Information Index, the number of private alleles, the expected heterozygosity, *R*_st_ and the estimation of gene flow. A principal coordinates analysis (PCoA) and an analysis of molecular variance (AMOVA) were also performed. Furthermore, we used a Bayesian approach to estimate the number of genetic clusters present in the whole sample. This model-based analysis was run with the software STRUCTURE version 2.3.3 (Pritchard *et al*. 2000), using a batch-oriented web programme package for construction of super matrices ready for phylogenomic analyses (Kumar *et al*. 2009). We ran 10 replicates for each *K* value ranging from 1 to 10 with a burn-in length of 50 000 followed by 500 000 iterations of each chain using the admixture model along with the assumption of correlated allele frequencies between groups (Falush *et al*. 2003). STRUCTURE then partitioned individuals of the sample according to the membership coefficient *Q*, that ranges from 0 (lowest affinity to the group) to 1 (highest affinity to a group), across *K* groups. Estimation of the best *K* value was conducted with STRUCTURE Harvester (Earl and von Holdt 2012) following the Evanno *et al*. (2005) method. The optimal *K* repetitions were permuted in Clumpp version 1.1.2 (Jakobsson and Rosenberg 2007), using the Greedy algorithm, with results graphically represented using Distruct version 1.1 (Rosenberg 2004). The population matrix is available at DEMIURGE (http://www.demiurge-project.org/) with digest code D-NMICR-98.

### Species distribution modelling

#### Modelling approaches

Since it is likely that the current distribution range on the studied islands is much reduced as a consequence of human activities (e.g. changes in land use and biological invasions), true absences are not available in this case. We thus opted to use modelling methods based on presences only, namely ecological niche factor analysis (ENFA) and maximum entropy modelling. Such an approach allows us not only to estimate the potential distribution and the habitat suitability for the species but also to identify the macroecological factors that might affect species distribution (e.g. altitude, climate, land use). The ENFA (Hirzel *et al*. 2002, 2006, 2007; Martinez *et al*. 2006; Hirzel and Le Lay 2008) provides smooth responses to environmental factors (Václavík and Meentemeyer 2012). This is desirable for modelling potential distributions, as models fitting complex responses may not accurately predict the distribution of species that are not at equilibrium. This approach was used successfully to model Azorean plant species, both invasive and native (Costa *et al*. 2012, 2013*a*; Moreira *et al*. 2014; Martins *et al*. 2015). Due to its wide application, Maxent was used for comparison (Phillips *et al*. 2004, 2006; Phillips and Dudík 2008).

#### Distribution data

The species presences (Fig. 1) recorded at the Atlantis data base (shape file format) were transformed into raster format at the same resolution as the ecogeographical variables (EGVs) for input in Biomapper (Idrisi raster format) and in Maxent (ASCI format).

#### Ecogeographical variables

As the number of presences was relatively low (<100), we followed Lomba *et al*. (2010) regarding the number of EGVs that should be used. We used three EGVs categories: climate, topography and land cover. Climatic variables were selected from the CIELO model (Azevedo 1996; Azevedo *et al*. 1999), a raster GIS environment with 100 m spatial resolution which models local scale climate variables relying on limited available data from synoptic coastal meteorological stations, and based on physical models that simulate the movement of air masses and their interaction with island topography [for more information, see http://www.climaat.angra.uac.pt or Azevedo (2003)]. We used the annual average of minimum, maximum, mean and range values of temperature (TMIN, TMAX, TM, TRAG), relative humidity (RHMIN, RHMAX, RHM, RHRAG) and precipitation (PMIN, PMAX, PM, PRAG). In addition, these climatic variables were submitted to a principal component analysis (PCA) as most of them were highly correlated. The principal components explaining more than 90 % of variance in the original variables were held and used alternatively. Those components, used as five alternative EGVs, corresponded to the first two components extracted from temperature (TPC1–2) and relative humidity variables (RHPC1–2), and to the first component extracted from precipitation variables (PCP). The topographic and land cover EGVs were acquired from the supporting data available in the CIELO model database, which matches the same spatial resolution of 100 m. To characterize the topography, we used the elevation (ELE) and the slope (SLP). Land cover was defined in six classes: (i) forest, (ii) natural vegetation, (iii) pasture, (iv) agriculture, (v) barren/bare areas and (vi) urban/industrial areas. We tested two different approaches for land cover data. The land cover classes were sorted in the foregoing order to define an ordinal land cover (OLC) variable, from ‘like forest’ (forest) to ‘unlike forest’ (urban/industrial areas). Moreover, distance variables were calculated for each land cover class (DLC1–6). Distance variables express the distance between the focal cell and the closest cell belonging to a given land cover class. In total, 26 EGVs were tested. A global model was calculated in Maxent including all available variables. Only variables providing more information to the model were kept for each of the following descriptors: elevation, temperature, relative humidity and rainfall. These four variable groups were differently combined with other physiographic descriptors: aspect, slope, flow accumulation and hill shade.

#### Modelling

Models were run with Maxent, and the best model was selected, based on the analysis of jackknife permutations and AUC (area under the curve). The same data were used to run the model in Biomapper. An R script was used to calculate the Boyce index and the Boyce curve, as well as to compare the habitat suitability maps obtained in the two methods, using the McNemar test (for further details on model validation and comparison, see Costa *et al*. 2012, 2013*a*, and references within). The best model was then changed to include one or two variables describing the distance to different types of land use. The best model was selected and compared as previously. The projection of the potential distribution of *V. dabneyi* in Faial Island was obtained using Maxent.

## Results

### Demographic analysis

*Veronica dabneyi* individuals usually grow linearly and horizontally, according to the procumbent nature of the stems. At Miradouro Craveiro Lopes we found 15 plants, with a stem length of 16–70 cm. Only 4 (27 %) showed inflorescences, ranging from 1 to 2, with 7.5–18 cm in height and 6–23 fruits per inflorescence (mean = 14.6, sd = 6.9). At Tapada da Forcada, we found 12 plants, with a stem length of 8–65 cm, and 5 seedlings. Only six (50 %) showed inflorescences, ranging from 1 to 15, with 8–21 cm in height and 8–31 fruits per inflorescence (mean = 19.1, sd = 5.8). At Madeira Seca (Corvo) we found 10 plants, with a stem length of 6–35 cm, and 1 seedling. Of those, 9 (90 %) showed inflorescences, ranging from 1 to 24, with 3–13.5 cm in height and 4–20 fruits per inflorescence (mean = 8.8, sd = 3.6).

In Flores the number of seeds per fruit ranged from 1 to 24 (mean = 10.4, sd = 6.3) while in Corvo, a larger variation was found (1–31 seeds, mean = 15.4, sd = 7.2). Seed diameter was similar on both islands (Flores: mean = 1.20 mm, sd = 0.13 mm; Corvo: mean = 1.09 mm, sd = 0.17 mm). However, the weight of 100 seeds ranged from 0.00049 g in Flores, to up to 0.00074 g in Corvo. Based on those values, we estimated seed production as 5870 ± 309.9 (mean ± se) seeds at Madeira Seca, 4969 ± 500.4 seeds at Tapada da Forcada and only 760 ± 76.6 at Miradouro Craveiro Lopes.

### Associated flora and invasive species

**Figure 2.**
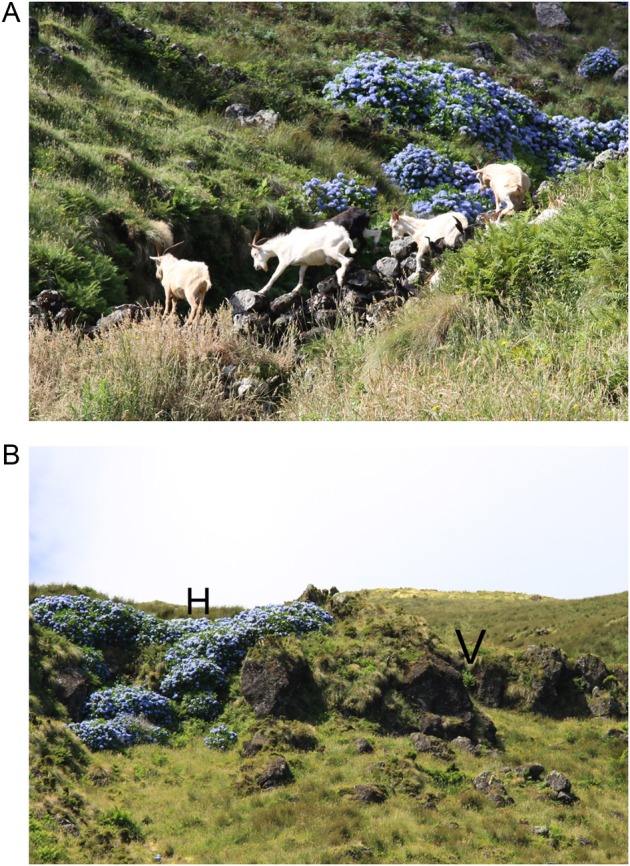
Two of the most relevant threats affecting *V. dabneyi* populations in the Azores: (A) feral goats free roaming and grazing very close to Madeira Seca in Corvo Island; (B) *Hydrangea macrophylla* clumps very close to a rock outcrop in Madeira Seca.

The flora closely associated with the presence of *V. dabneyi* included mostly other endemic taxa (Table 4). However, surrounding *V. dabneyi* populations, *Hydrangea macrophylla* (Thunb.) Ser. (Hydrangeaceae Dumort.), one of the most problematic invasive species in Flores and Corvo Islands (Silva *et al*. 2008), was found. In Flores, the largest populations were located along steep road sides. In Corvo, the population of Madeira Seca is established on vertical walls of a volcanic chimney. At this location, the main threats identified were the presence of feral goats (Fig. 2A) and the proximity of *H. macrophylla* clumps (Fig. 2B).

**Table 4.** Vascular plants associated with *Veronica dabneyi*, observed by Pereira *et al*. (2002) and by the authors.

Taxa	Family	Life form	Origin	Pereira *et al*.	Authors
*Blechnum spicant*	Blechnaceae	Hemicryptophyte	Native	X	X
*Calluna vulgaris*	Ericaceae	Chamaephyte	Native		X
*Centaurium scilloides*	Gentianaceae	Chamaephyte	Endemic		X
*Deschampsia foliosa*	Poaceae	Hemicryptophyte	Endemic	X	X
*Euphrasia azorica*	Orobanchaceae	Chamaephyte	Endemic		X
*Festuca francoi*	Poaceae	Hemicryptophyte	Endemic	X	X
*Frangula azorica*	Rhamnaceae	Phanerophyte	Endemic		X
*Hedera azorica*	Araliaceae	Phanerophyte (scandent)	Endemic		X
*Holcus rigidus*	Poaceae	Hemicryptophyte	Endemic	X	X
*Hypericum foliosum*	Hypericaceae	Phanerophyte	Endemic		X
*Ilex azorica*	Aquifoliaceae	Phanerophyte	Endemic		X
*Juniperus brevifolia*	Cupressaceae	Phanerophyte	Endemic		X
*Lotus pedunculatus*	Fabaceae	Hemicryptophyte	Introduced		X
*Luzula purpureosplendens*	Juncaceae	Hemicryptophyte	Endemic		X
*Lysimachia azorica*	Primulaceae	Chamaephyte	Endemic	X	X
*Myosotis azorica*	Boraginaceae	Chamaephyte	Endemic		X
*Picconia azorica*	Oleaceae	Phanerophyte	Endemic		X
*Rubia agostinhoi*	Rubiaceae	Chamaephyte	Endemic		X
*Rubus hochstetterorum*	Rosaceae	Phanerophyte (scandent)	Endemic		X
*Scabiosa nitens*	Caprifoliaceae	Hemicryptophyte	Endemic	X	X
*Selaginella kraussiana*	Selaginellaceae	Hemicryptophyte	Native	X	X
*Viburnum treleasei*	Adoxaceae	Phanerophyte	Endemic		X
*Woodwardia radicans*	Blechnaceae	Hemicryptophyte	Native		X

### Seed germination and seedling establishment

**Figure 3.**
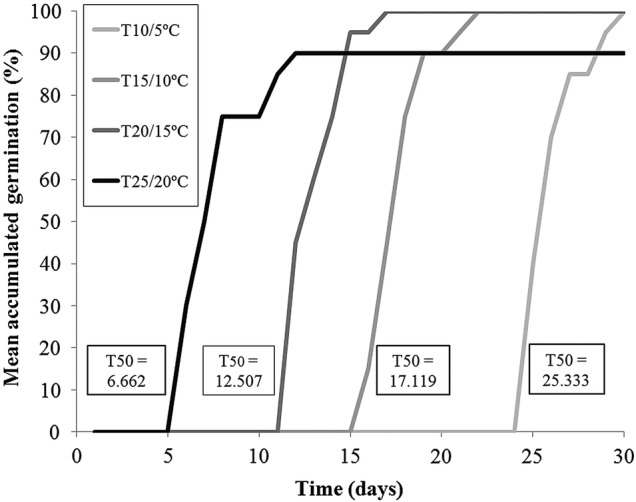
*Veronica dabneyi* accumulated seed germination curves obtained under 12 h of light and four alternating temperature regimes in Petri dishes in acclimatized chambers, after 30 days. Time for 50 % germination, in days, is shown for each treatment, based on the Gompertz model that was adjusted to each curve (all models with an *R*^2^ of >0.9).

The germination percentage of the seeds collected at Craveiro Lopes in July 2008 was high for all temperature regimes (90–100 %), with faster germination occurring at the higher ones (Fig. 3). However, seed batches lost viability after 2 years of storage at room temperature, with no germination in 2010. Seeds collected in June 2010 at Tapada da Forcada and at Madeira Seca did not germinate; however, they were most likely not mature at the time of collection.

### Population genetics

**Figure 4.**
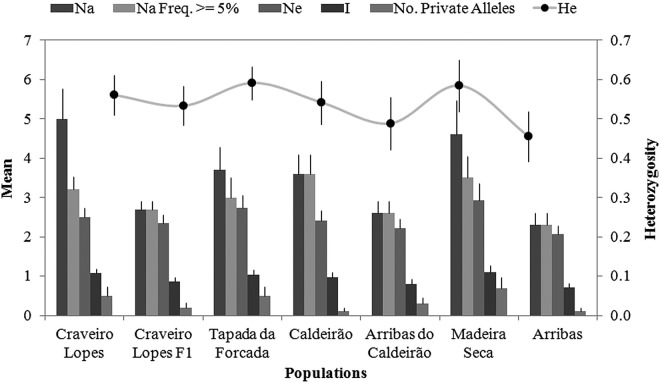
The mean allelic patterns across populations. Results of an analysis using GenAlEx on a total of 72 samples from 7 populations of *V. dabneyi* from Flores and Corvo islands (Azores) analysed with 10 microsatellite loci. Na, total number of alleles; Na (Freq ≥5 %), number of alleles with a frequency ≥5 %; Ne, number of effective alleles; I, Shannon's Information Index; No. Private Alleles, number of alleles unique to a single population. The line represents expected heterozygosity.

**Figure 5.**
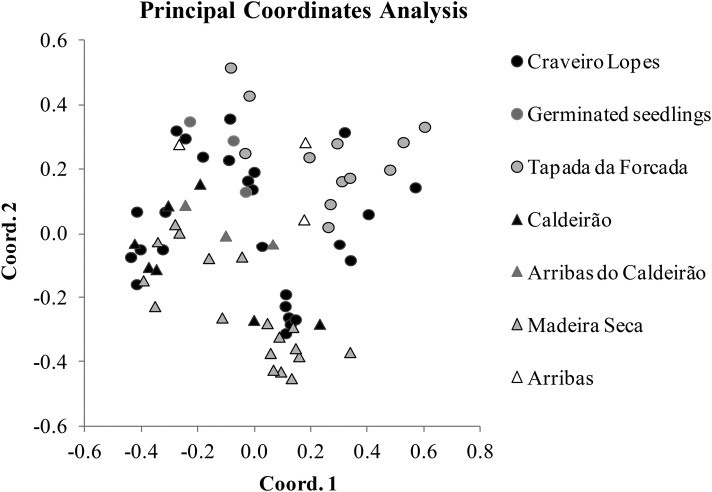
Graphic representation of the two first PCoA axes which explained 63.8 % of the detected genetic variation. Results obtained with GenAlEx for 10 microsatellite loci applied to 72 samples of *V. dabneyi* collected at six sites in Flores and Corvo islands, Azores. Germinated seedlings were derived from seeds obtained from Miradouro de Craveiro Lopes.

**Figure 6.**
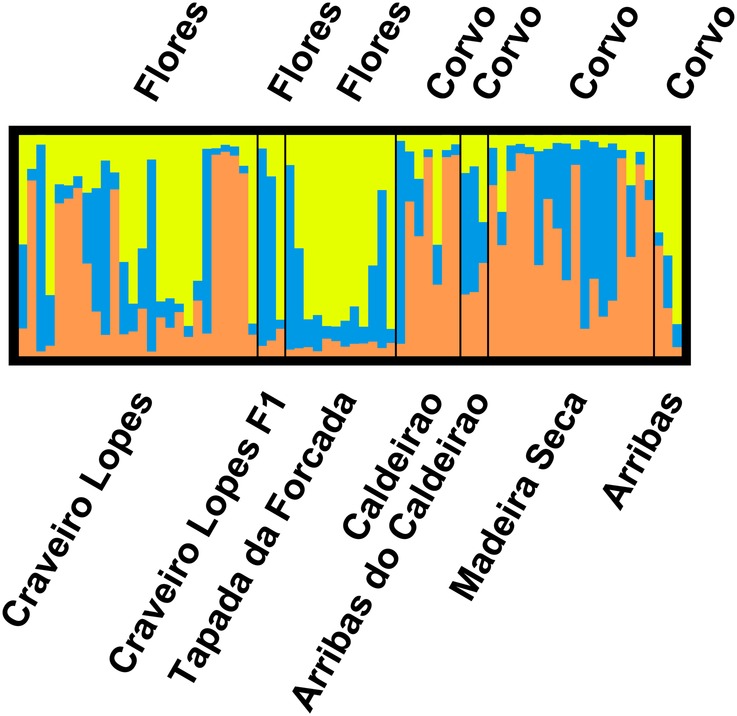
Results of the analysis of genetic clusters using STRUCTURE and DISTRUCT on a total of 72 samples from 7 populations of *V. dabneyi* from Flores and Corvo islands (Azores) analysed with 10 microsatellite loci. The number of genetic clusters was estimated at three, using a model including admixture.

A total of 72 samples from 7 populations were analysed at 10 microsatellite loci. The results of the AMOVA indicated that the majority of the genetic variation was found within the populations (98 %), and only a small portion among populations (2 %). The permutation test showed that the *R*_st_ value (0.033) was not significant (*P* = 0.160) with an estimated gene flow of 7.249. Also, the *R*_it_ value (−0.451) was significant and negative (Prand ≥ data, 1), revealing a lack of genetic population structure. The inbreeding coefficient *R*_is_ (−0.501) was significant and negative (Prand ≥ data, 1). Diversity patterns were similar across the sampled populations (Fig. 4). The average number of alleles ranged from 2.5 to 5, with the average number of private alleles per population below 1 (Fig. 4). Expected heterozygosity was somewhat homogeneous across the sampled populations, ranging from 0.46 to 0.59 (Fig. 4). The results obtained with the PCoA and STRUCTURE (Figs 5 and 6) showed a considerable degree of admixture, with three genetic clusters identified and represented at various degrees in all the sampled populations. Miradouro Craveiro Lopes individuals seem to encompass all the genetic variability found in the other populations of both Flores and Corvo (Figs 5 and 6). The F1 generation (i.e. resulting from seed germination) obtained from Miradouro Craveiro Lopes population showed genetic patterns compatible with those obtained in the source population (Fig. 6). The Corvo populations showed slightly different genetic patterns, at the exception of the Arribas population that is similar to those found in Flores (Fig. 6).

### Species distribution modelling

**Figure 7.**
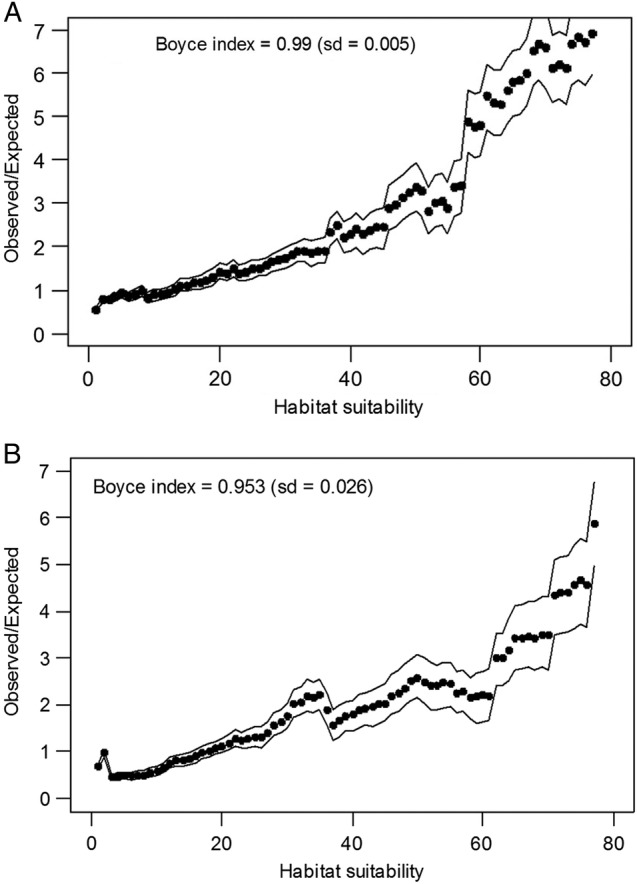
Boyce curve and Boyce index for the best distribution models for *V. dabneyi*, calculated using six EGVs in Biomapper: (A) the model with climatic and physiographic variables only (elevation, hillshade summer, annual rain fall, relative humidity annual range, annual mean temperature, slope); (B) the model including land-use information (elevation, annual rain fall, annual mean temperature, slope, distance to the nearest cell with agriculture, distance to the nearest cell with abandoned/unused land).

**Figure 8.**
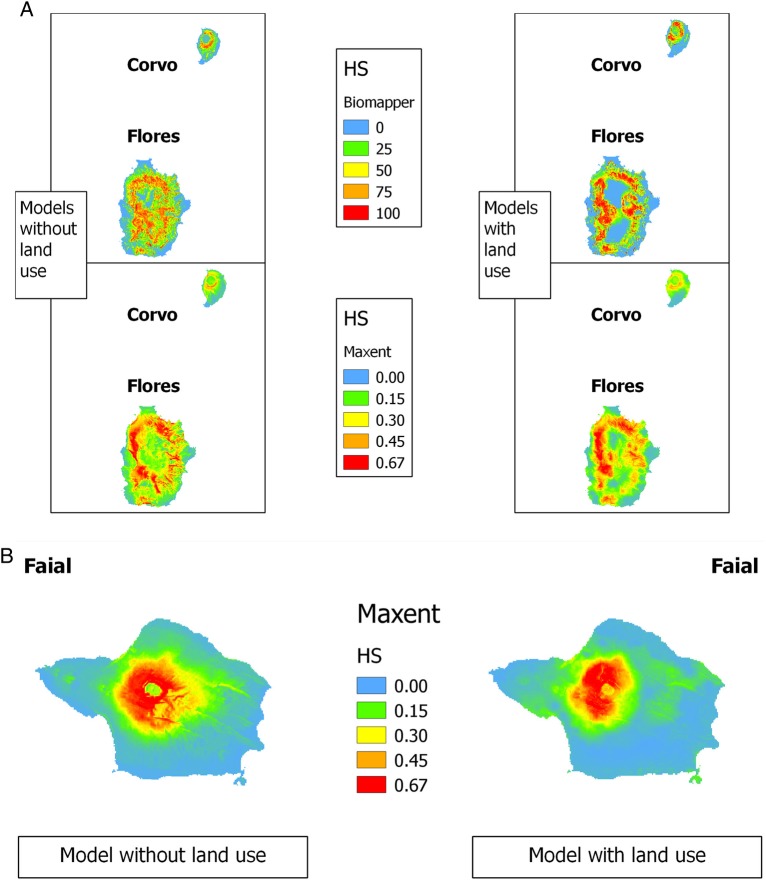
Habitat suitability maps for *V. dabneyi* in Corvo, Flores and Faial islands, calculated using six EGVs. (A) Models derived using Biomapper and Maxent for Flores and Corvo islands, either using physiographic and climatic variables only, or including land-use information also. (B) Projection of the potential habitat on Faial Island calculated using Maxent. Habitat suitability values were approximately divided according to the respective quartiles.

The best distribution model found in Maxent (AUC = 0.840) also corresponded to a good model in Biomapper (total information explained = 0.852, for the first three factors; Boyce index = 0.990) (Fig. 7A). Although there was a slight increase in the AUC (0.865) and in the total information explained (0.871 for the first three factors; Boyce Index = 0.953), according to the shape of the Boyce curve, the best model including land-use information was not as good as the model based on physiographic and climatic data alone (Table 5, Fig. 7B). When considering only the cells with habitat suitability above the third quartile, the McNemar test did not show significant differences between the results obtained with Maxent and Biomapper (model without land-use data, *χ*^2^ = 0.264, *P* = 0.607; model with land-use data, *χ*^2^ = 0.250, *P* = 0.617; Fig. 8). Likewise, marginality and specialization gave similar results for both models (marginality 0.426 and 0.349; specialization 1.108 and 1.144). The low marginality indicated that the habitat actually occupied by the species is similar to the average conditions of the available habitat. The relatively low specialization suggested that the conditions for species occurrence were not narrow. The analysis of the score matrix for the best models showed a positive link with elevation and slope, which affect the niche of the species, a negative link with the temperature, a positive link with rainfall and a negative association with high relative humidity ranges. It also demonstrates a negative association with the distance to uncultivated areas and a positive association with the distance to cultivated areas (Table 5).

**Table 5.** Results of modelling *Veronica dabneyi* distribution in Flores and Corvo islands using ENFA (Biomapper). Score matrix of each variable for the first three extracted factors for best models including or not, information regarding land use.

EGV	Factors		
	1	2	3
Model without land use			
Elevation	0.50	0.58	−0.04
Hillshade Summer	−0.32	0.18	−0.57
Slope	0.28	0.25	−0.23
Mean annual temperature	−0.47	−0.17	−0.46
Annual rainfall	0.28	0.00	−0.57
Relative humidity annual range	−0.52	0.74	0.30
Model with land use			
Elevation	0.61	0.30	0.64
Mean annual temperature	−0.57	0.38	0.50
Annual rainfall	0.34	−0.01	0.08
Distance to agricultural land	0.21	0.45	−0.52
Distance to uncultivated land	−0.13	0.74	0.24
Slope	0.34	0.12	0.02

## Discussion

### Demographic analysis

The heterogeneity of plant sizes in the three study populations suggests that there is frequent recruitment of new individuals from seed. This is more evident at Tapada da Forcada (Flores), a population with an almost uniform distribution of life stages, including 30 % seedlings and 35 % seed-producing individuals. At Madeira Seca (Corvo), more than 80 % of the individuals were reproducing, whereas at Miradouro Craveiro Lopes individuals were usually large but produced fewer inflorescences, resulting in a much lower total seed set. The existence of larger individuals with low number of inflorescences might be associated with regular brush cutting (see below). The shortest plant with an inflorescence measured 6 cm, although fruit production was more common in individuals with a length of ≥12 cm. This might imply that seed-producing plants can be relatively young. Despite the considerable variations in plant size and number of inflorescences, the estimated seed set for two of the studied populations was relatively high. The very small size/weight of the seeds, rapid germination, absence of dormancy and relatively fast decline on viability suggested that persistence as seed bank should be low (Yu *et al*. 2007), and this was confirmed in our study by the absence of germination following 2 years of storage at room temperature.

### Habitat and threats

*Veronica dabneyi* was found to be mainly associated with other endemic species. The conservation of native vegetation cover is thus of the utmost importance for the preservation of this species. It grows at sites with low vegetation cover mostly dominated by *Festuca francoi* and *Deschampsia foliosa* Hack. (Poaceae), generally found in forest openings and at steep locations such as waterfalls or road side slopes (Sjögren 1973). Therefore, the conservation of this type of vegetation, by avoiding changes in steep areas, will also be necessary. While populations of *V. dabneyi* were generally recorded on steep locations, the largest ones were found along steep road sides. On Flores, the population found at Miradouro Craveiro Lopes is located in front of a viewpoint, where brush cutting is regular. Changes in the road or at the top of the road side slope might affect this population. A similar situation was found at Tapada da Forcada, where the existing population occurs in a very steep slope, along the road. At some sites, the presence of *H. macrophylla* is a threat that should not be ignored, since this plant invader develops pure stands, and can outcompete all other plant species (Silva *et al*. 2008). In the Azores (Costa *et al*. 2013*b*), as in similar island groups and in many regions worldwide, plant invaders are commonly found within protected areas (see Foxcroft *et al*. 2013) and hydrangeas are even actively planted by Azorean farmers and the forest department as ‘green fences’. The hydrangea shrub can form impenetrable stands and stop cattle from falling down cliffs. These cliff-top hydrangea plantations spread throughout the cliff sides, outcompeting endemic species that survive in these inaccessible refugia. Hydrangea clumps are very close to several *V. dabneyi* populations both in Flores and Corvo and might overgrow those populations in the near future. Thus, *H. macrophylla* clumps that are detected near *V. dabneyi* occurrences should be considered as priority targets for removal/control measures, and its plantation on cliff tops should be banned. Instead, barbed wire fences should be preferred to keep cattle away from dangerous areas with no significant impact on natural vegetation. Other important herbaceous invasive species like *Hedychium gardnerianum* Ker Gawl. (Zingiberaceae Martinov) (Silva *et al*. 2008) should also be monitored at *V. dabneyi* sites.

Although direct evidence of *V. dabneyi* consumption by goats, cattle and rabbits is rare, its distribution at Caldeirão in Corvo Island, mostly on steep, rocky outcrops, suggests a possible retreat from areas more fully accessible to herbivores (Milchunas and Noy-Meir 2002). During our visits we found that cattle were free to roam at Caldeirão, even on sensitive vegetation like peat bogs that are important systems for water absorption and retention, and that goats were generally feral or unherded. This poses a big threat not only to *V. dabneyi* but also to other herbaceous endemic species (Houston *et al*. 1994; Silva *et al*. 2008) like *Tolpis azorica* (Nutt.) P.Silva (Asteraceae Bercht. & J.Presl) or *Euphrasia azorica* H.C.Watson (Scrophulariaceae Juss.), which are eaten in all accessible places. In the Azores it was not yet possible to use herbivore-exclusion plots, but this approach was used in the Canary Islands, showing that herbivores exert a strong negative effect on plant establishment, demanding the implementation of conservation measures, such as large fenced areas, control activities and eradication (Garzón-Machado *et al*. 2010). Meanwhile, the necessary changes in herbivore management clearly include a societal component, involving stakeholders that are not directly linked to conservation (e.g. hunters, farmers, the agriculture services).

### Seed germination and seedling establishment

The fastest germination treatment occurred under the 25/20 °C temperature regime (T50 = 6.7 days), germination reaching 90 %. The rate of 100 % germination was obtained with a temperature regime of 20/15 °C, given the second best T50 (12.6 days). Similarly, high germination percentages were also obtained for the related *Veronica arvensis* L. (King 1975). For conservation purposes, seeds should be collected after full development of the fruits which generally occurs by mid-July, and promptly sown. It was possible to grow seedlings obtained from germination in the laboratory and to plant them at the site of origin. For the first 2 years, the plants were monitored every 6 months and it was found that the seedlings planted next to the mother population survived at a rate of ∼50 %. Some early mortality due to transportation/establishment, and later mortality associated with roadside maintenance activities, was found. Furthermore, the genetic analysis of the germinated seedlings showed no considerable reduction in the genetic variability. Thus, the use of seeds might be a valuable option in a future recovery plan, in cases where population reinforcement might be preferred.

### Population genetics

Contrary to our initial expectations that were based on the existence of small and possibly isolated populations, the levels of genetic diversity found in the populations were relatively high, as was the level of genetic admixture, with no evidence of inbreeding. Expected heterozygosity was similar to that found for other Azorean herbaceous endemic taxa (Dias *et al*. 2014), which are much more common than *V. dabneyi*. The concentration of most of the genetic variation within populations was similar to patterns found for some of the endemic trees of the archipelago (Martins *et al*. 2013; Moreira *et al*. 2013). In a study using amplified fragment length polymorphism (AFLP) markers with *Veronica hederifolia* L., a European species invasive in China, high levels of genetic diversity were also found, and most of the total variance was attributed to that within (76 %) rather than between the populations (24 %) (Wu *et al*. 2010). Regarding *Hebe speciosa* (R.Cunn. ex A.Cunn.) Andersen (Plantaginaceae), a threatened endemic New Zealand shrub, using AFLP markers it was found that there is negligible contemporary gene flow, and that some of the populations exhibited extremely low genetic diversity (Armstrong and De Lange 2005). In *V. dabneyi*, high levels of gene flow and genetic admixture among the sampled populations presently impede clear population differentiation, even between Flores and Corvo. It should be noted that flower morphology of *V. dabneyi* suggests an entomophily syndrome (Garnock-Jones 1976): many-flowered inflorescence well above the level of the leaves; the background colour of the corolla is generally lavender with darker guide marks radiating from this ring to the surrounding corolla lobes, especially the posterior lobe. The related *Veronica chamaedrys* L. is mainly pollinated by hoverflies and short-tongued bees. Other possible pollinators include the Ichneumonidae (Garnock-Jones 1976). We frequently observed a range of Diptera species on *V. dabneyi* flowers, especially syrphid flies, dung flies and small, unidentified dipterans. Even though they probably do not regularly cross the channel between Flores and Corvo, they could easily be blown between islands in stormy weather, thus transferring pollen from island to island. Alternatively, the present population genetic structure might be the result of a bottleneck effect, consequence of the fragmentation of a previously wider species distribution range that was reduced due to land-use changes (Arenas *et al*. 2012). In fact, our modelling results do suggest that the potential distribution of *V. dabneyi* could have been wider in the past. Still another possibility is that the population at the Miradouro Craveiro Lopes, which shares most of the sampled genetic diversity, could be the result of a human translocation of plant material, although written records are not available. In this regard, although seed germination might be a good option to reinforce the most depauperate populations, we do not support translocation of plants between different populations and, particularly, between different islands. The species seems to comprise one global meta-population with gene flow among populations, but there is no reason for translocation, which would be an artificial intervention in the natural gene flow patterns. Meanwhile, the occurrence of natural gene flow might be viewed as a positive factor for the conservation of *V. dabneyi*, ensuring the transfer of genetic information among populations, and avoiding extreme cases of inbreeding depression (Li *et al*. 2012).

### Species distribution modelling

The macroecological factors modelled showed that, at this scale, *V. dabneyi* is neither a marginal nor a highly specialized species. Both modelling approaches used, ENFA and Maxent, provided similar results, showing that its potential distribution largely coincides with the intermediate elevation zone in Flores and with the Caldeirão zone in Corvo. While ENFA had already been used in the Azores to model invasive and native trees (Costa *et al*. 2012, 2013*a*; Moreira *et al*. 2014; Martins *et al*. 2015), this is a first result devoted to modelling the distribution of a rare plant in the Azores, further supported by the agreement obtained with Maxent. *Veronica dabneyi* was shown to prefer intermediate elevations, high slopes, relatively low temperature, high rainfall and small variations in relative humidity. Land-use data did not increase the model quality in a sensible way but suggested the existence of a negative correlation with agricultural land. This largely coincides with the type of habitat known for *V. dabneyi*, suggesting that microenvironmental factors like vegetation cover at a specific location or the presence of a rock outcrop might also be relevant for its establishment (Batik *et al*. 1992; Svenning 2001; Crain *et al*. 2014). It should also be stressed that, as stated above, *V. dabneyi* is mostly found associated with other native and endemic plants at sites with relatively low stature vegetation, which is frequently found at steep locations (e.g. volcanic craters, road side slopes). However, even those sites are not completely protected from herbivores (e.g. goats, rabbits) or human disturbance (e.g. roadside maintenance). Interestingly, the model based on Flores and Corvo occurrence data correctly predicted that *V. dabneyi* would have adequate habitat at the Faial Caldeira, in agreement with the previous record (Cunha and Sobrinho 1939).

### Conservation measures

Since the species shows a considerable degree of genetic diversity, high seed production and high germination rate, conservation measures should be devoted to: (i) monitoring of natural populations to detect possible changes associated with human impacts; (ii) effective management of herbivores, especially feral goats, in Corvo and Flores Island Natural Parks, delimiting areas from which cattle should be prohibited and feral goats removed and (iii) the use of population circumscribed seed germination and seedling growth to recover the most depauperate populations. Eventually, more field work should be directed to Faial Island, particularly in the whole Caldeira area, in order to detect a potentially still existing *V. dabneyi* population on that island. We hope that this study will stimulate the development of a scientifically based recovery plan for this species, while serving as a model to similar studies devoted to other rare or endangered endemic plant species worldwide.

### Towards a more holistic approach to research in plant conservation

Although it is a common approach to dedicate attention to specific areas involved in plant conservation, we consider that a more holistic approach, devoted to multidisciplinary studies of endangered plants, should serve as basis for designing management or recovery plans. The latter are an important gap in the Azores where conservation efforts dedicated to endemic plants do not follow integrated recovery plans, contrary to the situation, for example, in the Canary Islands (BOC 2012). In fact, more than 500 000 native plants are produced annually in the Azorean nurseries but their use does not follow approved recovery plans or strategies.

Why should conservation research follow a more holistic approach?

First, the absence of phylogenetic/systematic revues dedicated to endemic species might lead to erroneous conservation decisions or to the lack of action where it is needed. It was recently found that the most endangered plant in the Azores, known only from one location, is in fact an introduced species (Schaefer *et al*. 2011). In contrast, for several native genera, the number of taxa present in the islands is still unclear and often underestimated as shown by two recent studies that discovered overlooked endemic taxa with specific distribution patterns (Bateman *et al*. 2013; Moura *et al*. 2015). At another level, accumulated evidence for the population genetics of endemic trees in the Azores shows that levels of genetic diversity, and patterns of population structure, vary considerably among the evaluated taxa (Silva *et al*. 2011; Martins *et al*. 2013; Moreira *et al*. 2013; Moura *et al*. 2013), demanding a detailed study per taxon. Moreover, the absence of long-term monitoring data will not only preclude the evaluation of recovery programmes (Godefroid *et al*. 2011), but also impede a sound evaluation of conservation status, which has then to be determined based on distribution areas and not on observed population trends (Moreira *et al*. 2014; Martins *et al*. 2015). Monitoring is also linked to other relevant factors such as the detection of high mortality rates associated with herbivore pressure, making the propagation of high numbers of individuals an almost complete loss if no measures are taken to control predation (Donlan *et al*. 2003; Garzón-Machado *et al*. 2010). In the future, other aspects such as climate change will have to be integrated in long-term management or recovery programmes, making modelling approaches fundamental tools to support decision making (Fordham *et al*. 2012).

This more holistic approach can be accomplished by evaluating conservation status and possible management actions, based on a series of previous publications devoted to the target species (e.g. Moreira *et al*. 2014; Martins *et al*. 2015), or by developing multidisciplinary projects from the onset of the research programme, like it was done in the present paper.

Undoubtedly the need to address different aspects involved in the assessment and restoration of endangered plants arises directly from the Global Strategy for Plant Conservation, namely from its Objective 1 (*Plant diversity is well understood*, *documented and recognized*, CBD 2012). In our view, the different targets of this strategy will only be implemented if holistic approaches to research in plant conservation are effectively implemented in the near future.

Multidisciplinary studies like the one presented here, although sometimes longer, would increase the success of recovery and long-term maintenance of rare species, therefore improving the outcome of conservation investment, besides being a more powerful tool to halt plant extinctions.

## Sources of Funding

This research was supported by project VERONICA, funded by the Azorean Government, and by project DEMIURGO, funded by MAC-TCP.

## Contributions by the Authors

L.S. participated in sampling, performed species distribution modelling, conceived the idea for the paper and wrote the first draft; M.M., E.F.D. and J.S. were involved in the genetic analysis; M.M. performed the germination tests; E.B.A. provided climatic data; H.S. participated in field work. All authors helped revise the manuscript.

## Conflict of Interest Statement

None declared.
